# The reliability of pseudoneglect is task dependent

**DOI:** 10.1016/j.neuropsychologia.2020.107618

**Published:** 2020-11

**Authors:** A.G. Mitchell, J.M. Harris, S.E. Benstock, J.M. Ales

**Affiliations:** a School of Psychology, Philosophy & Language Sciences, The University of Edinburgh, UK; b School of Psychology & Neuroscience, The University of St Andrews, UK

**Keywords:** Pseudoneglect, Attention, Perception, Reliability

## Abstract

Bisection tasks that require individuals to identify the midpoint of a line are often used to assess the presence of biases to spatial attention in both healthy and patient populations. These tasks have helped to uncover a phenomenon called pseudoneglect, a bias towards the left-side of space in healthy individuals. First identified in the tactile domain, pseudoneglect has been subsequently demonstrated in other sensory modalities such as vision. Despite this, the specific reliability of pseudoneglect within individuals across tasks and time has been investigated very little. In this study, we investigated the reliability of response bias within individuals across four separate testing sessions and during three line bisection tasks: landmark, line bisection and tactile rod bisection. Strong reliability was expected within individuals across task and session. Pseudoneglect was found when response bias was averaged across all tasks, for the entire sample. However, individual data showed biases to both left and right, with some participants showing no clear bias, demonstrating individual differences in bias. Significant, cross-session within-individual reliability was found for the landmark and tactile rod bisection tasks respectively, but no significant reliability was observed for the line bisection task. These results highlight the inconsistent nature of pseudoneglect within individuals, particularly across sensory modality. They also provide strong support for the use of the landmark task as the most reliable measure of pseudoneglect.

## Highlights

• Reliability of pseudoneglect was assessed across 4 sessions and 3 tasks.
• The landmark task was the most reliable test for pseudoneglect across sessions.
• Responses to line bisection and tactile rod were less reliable across sessions.
• Responses to different bisection tasks in the same individuals were not reliable.

## 1 Introduction

Spatial neglect presents as an extreme preference towards the right side of space after a stroke to the right-hemisphere (Halligan et al., 1991). One way to assess the presence and magnitude of spatial neglect is through line bisection tasks (Molenberghs and Sale, 2011), where patients are required to manually transect the midpoint of a line. Neglect patients tend to transect the line much further to the right than the true midpoint, highlighting a substantial rightward bias in spatial attention (Binder et al., 1992; Halligan et al., 1991; Reuter-Lorenz et al., 1990). However, neurotypical individuals typically transect the line further to the *left* of its true midpoint (Benwell et al., 2014; Learmonth et al., 2015; Manning et al., 1990; McCourt and Olafson, 1997; Nicholls and Roberts, 2002). This phenomenon is known as pseudoneglect and has been repeatedly identified among the general population (Brooks et al., 2016; Friedrich et al., 2018; Jewell and McCourt, 2000) and is thought to be a result of a slight, lateralised bias to spatial attention (Heilman and Van Den Abell, 1979; Kinsbourne, 1970). Knowledge of cause and contributions of pseudoneglect can help to aid our understanding of spatial attention in neurotypical individuals as well as disorders of attention such as spatial neglect.

Two related hypotheses suggest pseudoneglect is due to imbalances in the orienting of spatial attention across left and right hemispheres of the brain. The Right-Hemisphere Dominance hypothesis (Heilman and Van Den Abell, 1979), describes pseudoneglect as a consequence of dominant orienting mechanisms for attention in the right-hemisphere, leading to an over-representation of the left, contralateral side of space. The Interhemispheric Competition hypothesis suggests that each hemisphere competes for the dominance of spatial attention (Kinsbourne, 1970, 1987), and biases in attention to each hemifield are dependent on the activation within each hemisphere. Despite slight differences, both theories suggest that underlying biases in spatial attention are present among the majority of individuals. Therefore, understanding the prevalence and reliability of pseudoneglect in the healthy population can potentially improve our understanding of human perception and attention.

If attentional mechanisms are responsible for pseudoneglect, performance in bisection tasks should be relatively stable. However, a systematic review by Jewell and McCourt (2000) highlighted how left-ward biases in bisection tasks appear to be dependent on a number of factors such as line length (Rueckert et al., 2002), age (Bradshaw et al., 1987; Failla et al., 2003; Fujii et al., 1995; Schmitz and Peigneux, 2011) and line position (Luh, 1995a; McCourt and Jewell, 1999; Milner et al., 1992; Reuter-Lorenz et al., 1990). Error in bisection tasks is also dependent on the egocentric position of the line: pseudoneglect is reduced for lines that are further away from the participant (Varnava et al., 2002). Moreover, a considerable amount of between participant variability in bisection tasks has been reported (Brooks et al., 2016; Learmonth et al., 2015; Luh, 1995; Manning et al., 1990).

There are three classic variations of the bisection task, each involving different perceptual mechanisms. The first bisection task used to identify a leftward bias in healthy individuals was tactile rod bisection. Blindfolded participants were found to significantly bisect a centrally aligned rod further to the left (~0.6 cm) of centre using both their left and right index fingers (Bowers and Heilman, 1980). However, by far the most frequently used tasks to identify both pseudoneglect and spatial neglect since then have been the landmark (Milner et al., 1993) and line bisection (Axenfeld, 1915) tasks. The landmark task is a perceptual alternative to line bisection and requires participants to identify which side of a pre-bisected line is perceived as longer (Harvey et al., 1995; Milner et al., 1993). In this task, classic pseudoneglect is represented as identification of the subjective midpoint of the line (point of subjective equality) as further to the left than the true midpoint (Bultitude and Aimola Davies, 2006; Jewell and McCourt, 2000; Learmonth et al., 2015; Milner et al., 1993). Left-ward biases are present in all three tasks using tactile, visual and visuomotor mechanisms and, as shifts to spatial attention are also known to affect more than one sensory modality (Driver and Noesselt, 2008; Driver and Spence, 1998; Farah et al., 1989; Spence and Driver, 1998), pseudoneglect should be reliable across modalities.

Indeed, pseudoneglect is present in a wide range of different tasks (Brooks et al., 2016; Learmonth et al., 2015; Luh, 1995b; Nicholls and Roberts, 2002). A significant left-ward bias is observed at the group level during the perceptual landmark task (Benwell et al., 2013; Learmonth et al., 2015; Milner et al., 1992, 1993), visuomotor line bisection (Bradshaw et al., 1986; Learmonth et al., 2015; Luh, 1995a; McCourt and Jewell, 1999), tactile rod bisection (Bowers and Heilman, 1980; Sampaio and Chokron, 1992; Sampaio and Philip, 1991), mental imagery (Brooks et al., 2014; Longo and Lourenco, 2010; McGeorge et al., 2007) and chimeric face judgements (Failla et al., 2003; Luh, 1995). However, very few studies have investigated the reliability of response bias to typical pseudoneglect tasks.

One study assessed response bias in a range of different visual pseudoneglect tasks within individuals, across two separate testing sessions (Learmonth et al., 2015). Inter-rater reliability was found across two testing sessions for both the landmark and line bisection tasks. However, response bias to each task was assessed over only two testing sessions in this study. As biases to spatial attention are known to be affected over a longer period of time (Hausmann, 2005), to be able to argue whether individuals have a stable bias to spatial attention, the presence of pseudoneglect needs to be assessed more extensively over a longer time period. Moreover, somewhat surprisingly responses within individuals to different tasks did not correlate (Learmonth et al., 2015). This indicates that there is very little inter-task reliability for pseudoneglect tasks such as landmark, line bisection and visual detection, however these tasks tap into visual and visuomotor domains only and do not clearly transcend different sensory modalities.

Evidence for the reliability of pseudoneglect across sensory modality is unclear, although research suggests poor reliability across tasks (Brooks et al., 2016; Learmonth et al., 2015) and high reliability within individuals (McCourt, 2001), studies that directly compare response bias to tactile, visual and other pseudoneglect tasks have produced mixed results (Brooks et al., 2016; Hughes et al., 2004; Learmonth et al., 2015; Luh, 1995a). Understanding whether biases extend across different sensory modalities is particularly important when it comes to bisection tasks, as they are typically used interchangeably within the pseudoneglect, spatial neglect and attention literature (Cavézian et al., 2012; Cicek et al., 2009; Fink et al., 2001; Jewell and McCourt, 2000). There is currently no study, to date, that has investigated individual responses to different bisection tasks that recruit different sensory modalities.

This study aims to provide an in-depth analysis of the reliability of response bias using different bisection tasks across multiple sessions and sensory modalities. Here, three different bisection tasks are used; landmark (Harvey et al., 1995; Milner et al., 1993), line bisection (Axenfeld, 1915) and tactile rod bisection (Bowers and Heilman, 1980) across four separate testing sessions. All three tasks require participants to recruit different sensory modalities to make judgements about line length. The line-bisection task uses both visual and motor information to make this judgement, the landmark requires purely visual information whilst tactile rod bisection uses tactile and motor information in the absence of vision. This experiment addresses two key questions: are bisection tasks within individual participants reliable across (1) session and (2) modality? Our hypotheses are two-fold. The first states that, if pseudoneglect is caused by either Right-Hemisphere Dominance or Interhemispheric Competition, then the size and magnitude of biases will be reliable over multiple testing sessions for all three bisection tasks. If, like spatial neglect, pseudoneglect is caused by supramodal spatial attention mechanisms, the second hypothesis states that biases will be present and consistent across different sensory modalities.

## 2 Materials & methods

### 2.1 Participants

29 healthy volunteers were recruited to participate in the experiment (22 female, mean age = 21.3, SD = 2.6, range = 11). All had normal or corrected to normal vision and were right-handed. As pseudoneglect is likely to be reduced in older age (Failla et al., 2003), we specifically recruited young adults aged between 18 and 35. Participants were not included in the study if their handedness score was below 40 on the Edinburgh Handedness Inventory (Oldfeld, 1971) and mean handedness was 86.8 (SD = 15.1, range = 59.0). All participants were recruited through the University of St. Andrews SONA participation scheme and reimbursed £5/hr for their time. Ethical approval was obtained from the University of St. Andrews Teaching and Research Ethics Committee (UTREC) and the experiment was conducted in accordance with the Declaration of Helsinki. Participants were required to complete the experiment four times, on four separate days at least 24h apart. Any participant that failed to complete all four sessions had their data removed from the study (N = 1). The remaining 28 participants completed all testing sessions. A total of 24 datasets were analysed, after four participants’ data were removed during the analysis stage.

### 2.2 Stimuli and apparatus

**Fig. 1:**
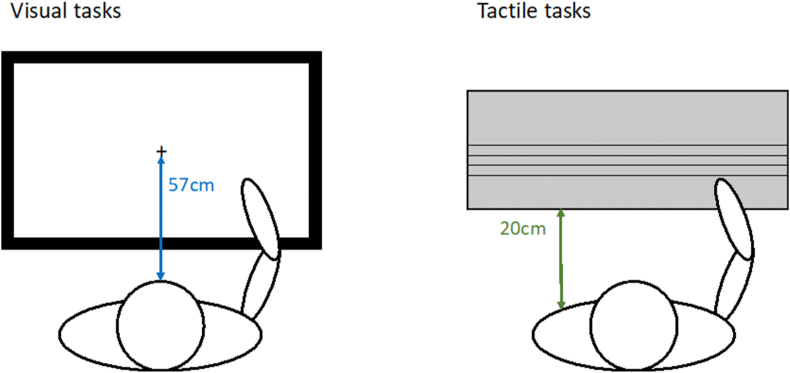
Layout of the visual screen-based task and tactile rod platform in relation to participant. Both the screen and platform were placed face up on the table in-front of the participant.

An Iiyama, ProLite, touchscreen monitor was used to present visual stimuli (active display = 46.5 × 24.5cm, resolution = 1920 × 1080). The experiment was performed using Psychtoolbox version 3.0.16 (Brainard, 1997) on MATLAB 64-bit R2018a. For the line bisection task, a capacitive stylus was used to record participants' responses, keyboard responses were used for the landmark task. All responses for landmark and line bisection were recorded using Psychtoolbox in MATLAB. The computer was positioned flat on the table at a viewing distance of 57cm in-front of the participant's body midline (Fig. 1).

For tactile rod bisection, black plastic rods (10mm diameter, lengths of 100, 200 and 300mm) were mounted on a platform (dimensions = 500 × 200 × 20mm) with two grooves (10mm apart, each 16mm at their widest point) running from one side to the other. Two metal, metric rulers were placed along the outer edge of each groove for accurate placement of the rod and to be able to measure hand position along the length of the rod to the nearest mm. Rods could be slid into the grooves and fixed in place by tightening the middle section in between the two grooves, using screws that were placed at either end. Hand placement for each trial was recorded manually by the experimenter. Both the touch-screen and the tactile-rod platform were placed flat and face-up on the table (Fig. 1) so that the set up for each task was in the same physical space relative to the participant (Bradshaw et al., 1985; Chokron and Imbert, 1993; Varnava et al., 2002).

### 2.3 Tasks

To avoid the effect of stimulus on response (Jewell and McCourt, 2000), line length and line position relative to the participant was controlled for in all tasks. To reduce effects of hand, the order of hand used (left, right) was evenly counterbalanced within sessions, across participants for line bisection. In tactile rod bisection hand used and starting hand position (left, right end of rod) were also evenly counterbalanced across participants, within sessions. To reduce order effects, the order of starting hand and side was also counterbalanced between testing sessions, within participants.

Each participant performed all tasks in the same order for all four sessions. For ease of testing, the visual tasks (line bisection and landmark) were run in the same block before or after the tactile rod bisection task. Order of tasks was counterbalanced across participants.

#### 2.3.1 Line-bisection

First, participants performed a touch-screen calibration to account for any error in viewing-angle and stylus position. Participants were asked to use the stylus to touch the centre of a cross ('+', 10 × 10mm) as accurately as possible at 12 different, sequential locations on the screen (12-point square grid). The calibration procedure was run twice, if the average error was over 5mm (20 pixels) then the experimenter re-ran the calibration until the error was < 5mm. The final calibration error for each participant was subtracted from the bisection error for the line bisection task, to account for individual differences in stylus position.

**Fig. 2:**
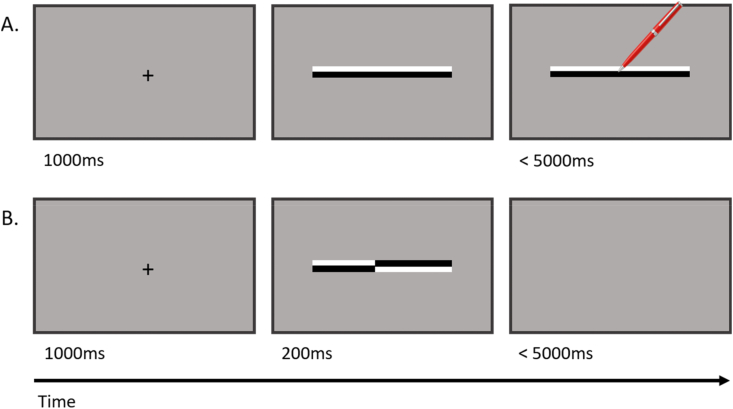
Illustration of line bisection (A) and landmark (B) tasks. (A) after a 1000 ms fixation cross participants were presented with a line and instructed to bisect the line using a stylus. (B) after a 1000 ms fixation cross a pre-bisected line was presented for 200 ms before disappearing. Participants had 5000 ms to respond in both tasks.

For the line bisection task, a black, central fixation cross was presented for 1000ms, after which a line of either 100, 200 or 300mm appeared (Fig. 2A). Lines were lines 9mm thick (50% white and 50% black) presented either in the middle of the screen (at the participant's midline) or 20 mm to the left or right. By shifting line position, participants could not always assume that the centre of the line was either the centre of the screen or aligned with their body midline. Participants were instructed to bisect a line, presented on the touchscreen, by reaching out and touching the perceived midpoint with a stylus (Fig. 2A). Once the participant touched the perceived middle of the line, the coordinate location of the touch was recorded, and the next trial began. If the participant failed to respond after 5s, the experiment automatically moved on to the next trial and the previous trial was removed from analysis.

For each block the participant completed 90 trials, 30 per line length and 30 per line offset, 45 per hand. Total time per block was 6–8 min and four blocks were completed, one in each testing session. 360 trials were completed across four sessions.

#### 2.3.2 Landmark task

For the landmark task, lines were adapted from (McCourt, 2001) and were 50% white and 50% black, at a particular point on the line the colours were reversed, creating two distinct line segments (Fig. 2B). The two segments could meet at either the middle of the stimulus (stimulus asymmetry of 0 mm) or 2, 4, 6, 8 and 10mm to the left or right. Each line segment met either at participant's body midline or 20mm to the left or right (to match line bisection), so the position of the participant's body could not affect response.

A black, central fixation cross was presented for 1000ms, followed by a pre-bisected line (100, 200 or 300mm in length) for 200ms. This duration allowed participants to view the length of each side of the line but restricted any opportunity to plan an eye-movement (Jewell and McCourt, 2000). Participants were asked to specify which side of a centrally-bisected line was longer using buttons on a keyboard (‘1’ – left side longer, ‘2’ – right side longer). After the line disappeared participants were encouraged to respond as quickly as possible (maximum response time of 5000ms). Participants completed 180 trials per session, 60 per line length, 15 for each left/right offset, and 30 for lines where each side was equal. There were double the number of trials for 0 mm stimulus asymmetry as this condition is the most sensitive at picking up biases in spatial attention. 720 trials were completed across all sessions, each block lasted around 5–6min, four blocks were completed, one for each testing session.

#### 2.3.3 Tactile rod bisection task

**Fig. 3:**
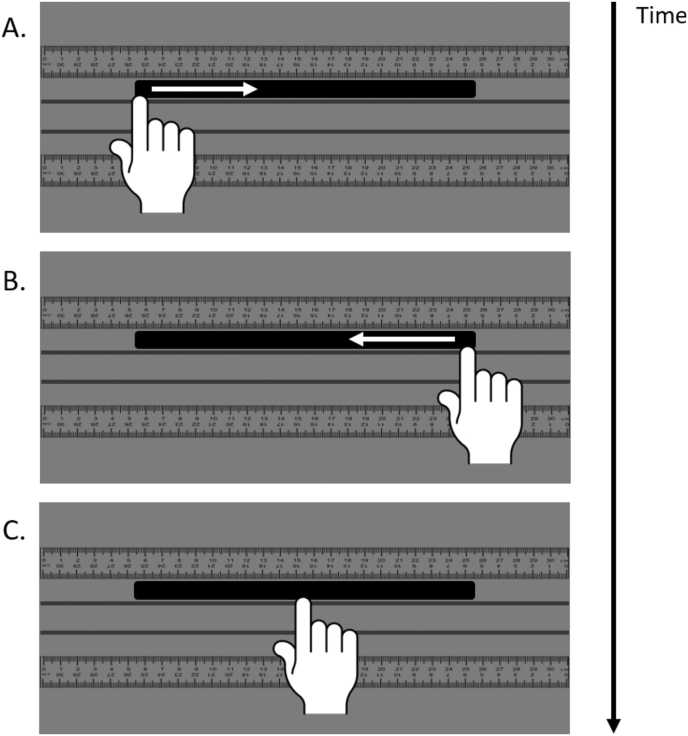
Tactile rod bisection task. (A) participants start at one side and scan in that direction to the opposite side, (B) participants scan the rod a second time in the other direction before finding the perceived midpoint (C).

At the beginning of each tactile session, participants placed their heads in a chin-rest to make sure that their body was always held in a straight, central position and were blindfolded so they never saw the length of rod used. For the experimenter to be able to identify where the participant transected the line to the nearest mm, a small, vertical line was drawn on the fingernail of each of the participant's index fingers. The experimenter placed a rod (either 100, 200 or 300mm) into the slot on the platform furthest away from the participant. Rod position was offset in the same manner as the line bisection task and landmark tasks. Participants were instructed to start scanning at one end with either their right or left index finger from either left or right hand-side (Fig. 3A) and fully scan the length of the rod before repeating the same action in the other direction along the rod (Fig. 3B). Finally, they were instructed to place their finger at the perceived midpoint of the rod (Fig. 3C). Once they had identified a perceived midpoint, the participant said ‘okay’ and the experimenter recorded the position to the nearest mm, relative to the participant's left-hand side of the rod, using the ruler aligned next to the rod. For example, if the participant bisected a 100mm rod 5mm to the left of centre, then the experimenter would record this value as 45mm. Once the experimenter had recorded the finger position, they indicated to the participant to take their hand back to their body midline. The experimenter adjusted the rod as needed, and the next trial began.

For ease of testing, each rod lengths were blocked. Rod length was changed every 18 trials within a session and order of rod length was counterbalanced within participants across testing sessions. To reduce order effects, starting rod length was counterbalanced across participants. Participants completed 54 trials per session, 18 per line length, 18 per line offset, 27 per starting position (right or left), 27 per hand. A total of 216 trials were conducted across all 4 sessions. For one session, a block lasted 15–20 min.

### 2.4 Data analysis

MATLAB R2018a was used for pre-processing, psychometric analysis, Cronbach's alpha (Salarian, 2020), tests of normality and t-tests. JASP 0.9.2 was used to conduct the repeated-measures ANOVAs.

#### 2.4.1 Individual-level analysis

##### 2.4.1.1 Line bisection

Line bisection error was defined as the perceived midpoint minus the physical line midpoint. Left-ward error is represented by negative values, rightward error is represented by positive values. Average bisection error across all three line-lengths was calculated for each session, for each participant.

##### 2.4.1.2 Landmark

**Fig. 4:**
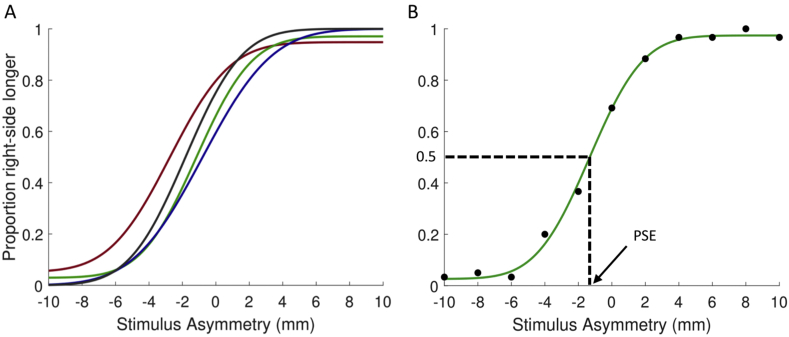
Example psychometric fits from the landmark task from one of the better participants. A) psychometric fit for each session. Session 1 = green, 2 = blue, 3 = red, 4 = grey. B) psychometric fit for data averaged across all testing sessions with example of how PSE is calculated. (For interpretation of the references to colour in this figure legend, the reader is referred to the Web version of this article.)

For the landmark task, the proportion of ‘right side longer’ responses were calculated for each shift to the left/right side of the line (0, 2, 4, 6, 8 and 10 mm) for each session. For example, a rightward shift (stimulus asymmetry) of 10 mm is expected to have 100% ‘right side longer’ responses as the right-hand side is unambiguously longer than the left, whilst for a stimulus asymmetry of 0 mm, stimulus responses are expected to be much closer to 50%. For each participant, a psychometric curve was fitted using a Cumulative Normal psychometric function from the Palamedes Toolbox (Fig. 4), (Prins and Kingdom, 2018). The slope and threshold were estimated using non-parametric bootstrapping. The guess rate (number of responses likely to be guesses) and lapse rate (number of incorrect responses due to task demands) were maintained as free parameters, the lapse rate was estimated using the jAPLE fitting scheme (Prins, 2012) and the guess rate was fixed to equal the lapse rate.

Psychometric functions were fitted to percentage right-side responses for each session (Fig. 4A). The percentage ‘right-hand side longer’ responses were also calculated for each line shift across all sessions and plotted as a function of stimulus asymmetry (where asymmetry = colour reversal point - physical centre of the line); a psychometric function was fitted to these data using the same parameter estimation methods as above (Fig. 4B).

After psychometric curve fitting, bisection error for each session was calculated using the point of subjective equality (PSE). The PSE identifies the point at which the observer considers each side of the line to be identical, this is the point along the x-axis where participants respond that the right-side of the line is longer than the left for half the proportion of trials (0.5 on the y-axis, Fig. 4B). For example, the PSE of the example dataset in Fig. 4B is −1.5 mm. Therefore, the left side of the line has to be 1.5 mm longer than the right for the participant to perceive the line as symmetrical. This participant has a right-sided bias, as, when both sides are equal, they perceive the left side of the line as shorter than the right.

To fit with the rest of the data, where left error = negative values and right error = positive, bisection error for the landmark task was defined as –PSE.

##### 2.4.1.3 Tactile rod bisection

For tactile rod bisection, bisection error was calculated in the same way as the line bisection task (perceived midpoint – physical line midpoint). Average bisection error across all three rod-lengths was calculated for each session, for each participant.

#### 2.4.2 Group-level analysis

To address the hypothesis that pseudoneglect is reliable across session, Cronbach's alpha was used to calculate an intra-class correlation coefficient (ICC, Bland and Altman, 1997; Shrout and Fleiss, 1979; Weir, 2005), across session and task modality. Intra-class coefficients are designed to compare reliability of repeated measures across more than two observations. This contrasts with the more commonly used Pearson's r correlation coefficient. Pearson's r is designed to measure the relationship between two things that are different units (e.g. centimeters & kilograms). The present study uses repeated measures across three tasks and four sessions; therefore, ICCs are appropriate to quantify reliability. There are multiple types of ICCs and choosing is not straightforward. We decided Cronbach's alpha (or ICC_c,k_) was appropriate by considering which ICC aligned with the design of the present study (for detailed guidance see McGraw and Wong, 1996; Weir, 2005). The main consideration is the difference between ICCs that quantify ‘absolute agreement’ (e.g. ICC_a,k_) compared with quantifying ‘consistency’ (e.g. ICC_c,k_). Absolute agreement requires values to be exactly the same and the correlation coefficient is reduced by the presence of task- and/or session-level group bias. Cronbach's alpha is a measure of consistency and removes task/session level biases. An α-value = 1 indicates perfect cross-session correlation within participants, representing perfect reliability (i.e. no measurement error) whilst values closer to 0 indicates test-retest values are not consistent. An α-value closer to −1 indicates a reversal in score agreement between measurements.

To address the hypothesis that pseudoneglect is reliable across modality, Cronbach's α was also used to assess consistency of bisection error across tasks within the same participants. Once again, pseudoneglect is predicted to be consistent across different spatial modalities, therefore a high Cronbach's α is expected. The bisection error was also averaged across participants in each task, and (as pre-registered, see *Section 3.5*) one-sample t-tests were run to assess the presence of pseudoneglect for the group in each task and each session. The use of multiple tests was corrected for using Bonferroni correction to ensure the family-wise error for observing a significant result is controlled at the .05 level. Using this correction, a significant p-value is equal to or below 0.05 multiplied by number of tests used. To detect pseudoneglect for each testing session, the adjusted alpha was 0.013 and for each task it was 0.017.

Finally, to investigate whether there were any clear significant differences between bisection error in different task modalities and across the four sessions a 3 × 4 repeated measures ANOVA was conducted with factors 1) task (landmark, line bisection, and tactile rod bisection) and 2) session (1, 2, 3 and 4). An interaction effect between task and session would indicate that the bias in different modalities is different in different sessions.

Exploratory analyses to check for effects of hand-used on the reliability of line and rod bisection error, and effects of colour reversal point on perceived subjective (PSE) midpoint in the landmark task, can be found in the Supplementary Materials. To summarise, we found biases were similar for right and left-hand in manual line bisection or tactile rod bisection across testing session, and for modality. For the landmark task, opposite patterns in colour reversal did not significantly affect PSE.

### 2.5 Data statement

All data, stimulus and analysis code have been made freely available on the Open Science Framework (OSF, https://osf.io/zebrd). Analysis and stimulus code are also available on GitHub (https://github.com/aleslab/MitchellEtAlPseudoneglect/).

### 2.6 Pre-registration details

The full protocol for this experiment was pre-registered on OSF on November 19, 2018 (https://osf.io/p6hnx) in advance of completing data collection and any data analysis. In this paper, we report the results of planned studies on cross-session and cross-modality reliability only, any further studies described in the pre-registration will be addressed and reported elsewhere. The methods and analyses reported here follow our planned pre-registration, with exceptions outlined below.

To identify the required sample size to detect reliable internal consistency using the Cronbach's alpha as well as a significant difference in the *t*-test, Monte-Carlo simulations (n = 1000, code available on OSF) were run using numbers extracted from pilot data. We found that 30 participants would provide >99% power to detect a Cronbach's α of at least 0.8 within testing sessions in the landmark, line bisection and tactile rod tasks. When data were collapsed across sessions, pilot data indicated that an N = 30 also provides at least 91% power to detect a significant effect of pseudoneglect in each task using a one-sample *t*-test. Due to time limitations and challenges in recruitment, only 29 participants took part in the study. One of those participants dropped out during data collection and planned analyses removed a further 4 participants. Data from 24 participants reduced predicted power to 98% for Cronbach's alpha, and to 89% for one-sample t-tests.

For final data analysis we revised the pre-registered outlier removal method, which we now consider to be too stringent, as it will reduce the presence of individual differences in response bias. Instead, outliers were identified if bisection error was >5 standard deviations away from the mean. One participant was removed from further analysis as they were identified as an outlier in all three bisection tasks. All other data analyses followed planned analysis in the pre-registration.

## 3 Results

Three participant's data were removed from analysis due to poor psychometric fits which made us unable to confidently predict their PSE. A further participant's data was removed because their line bisection and landmark error was as >5 SD from the group mean. The final number of total data sets analysed was 24.

Left-ward bisection error is represented by negative numbers, the rightward error by positive numbers. The mean bisection error for each session in each task, averaged across all participants, is presented in Table 1. An overall effect of pseudoneglect (left-ward bisection error) was found when responses were averaged across all sessions and all tasks (t = −3.33, *p* = .003, Cohen's *d* = −0.68).

**Table 1:** Mean (M) and standard deviation (SD) of bisection error for each testing session (1–4) and task. Units are mm.

	Mean					SD				
	1	2	3	4	All Sessions	1	2	3	4	All Sessions
Landmark	0.03	−0.46	0.04	−0.40	−0.20	1.89	1.88	1.61	1.67	1.35
Line Bisection	−0.36	−3.65	−2.10	−1.56	−1.92	7.25	4.85	2.81	5.02	2.73
Rod Bisection	−1.53	−1.51	−0.85	0.55	−0.84	5.15	4.81	5.14	4.02	3.31
All Tasks	−0.62	−1.87	−0.97	−0.47	−0.98	2.08	2.79	1.93	1.84	1.45

### 3.1 Hypothesis I: reliability over session

**Fig. 5:**
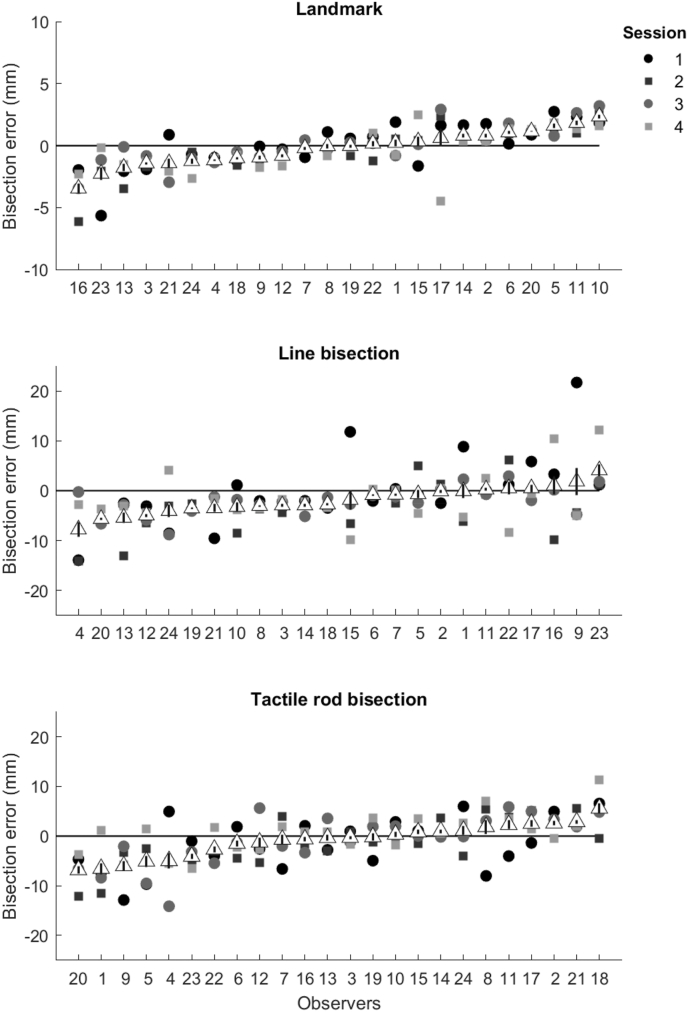
Individual bisection error for all 4 sessions in each task: landmark, manual line bisection, tactile rod bisection. Data are ranked by response bias from left (most negative) to right (most positive). Mean error for each individual in each task represented by the white triangle. Error bars show standard error of the mean.

Cronbach's α was used to assess the within-individual reliability of bisection error across session in each of the three tasks (Fig. 5). Strong reliability across testing sessions was observed for the landmark task (α = 0.80, *p* < .001, Fig. 5 top) and moderate reliability was found across sessions for the tactile rod bisection task (α = 0.63, *p* < .001, Fig. 5 bottom). However, more cross-session variance was observed for line bisection task (Table 1, Fig. 5 middle) and no significant reliability across testing sessions was observed (α = −0.11, *p* > .050).

**Fig. 6:**
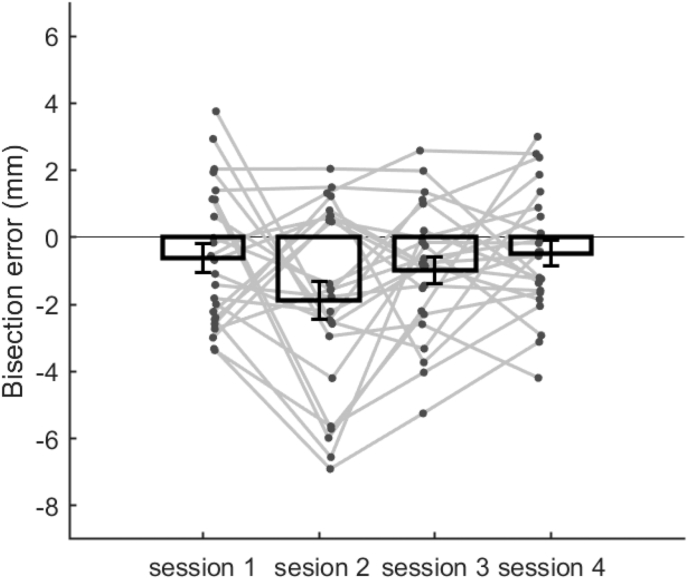
Summary of mean bisection error for each session (bars). Individual participant data for each task are displayed as grey dots. Error bars show standard error of the mean.

One-sample t-tests were also used to determine the presence of pseudoneglect in the entire sample for each testing session. A mean left-ward bisection error was found for all testing sessions (Fig. 6). However, errors were found to be significantly different from 0 in session two only (t_23_ = −3.29, *p* = .003), no other sessions showed a significant leftward response bias below the adjusted alpha level of 0.013.

Of all tasks, the landmark task produced the most reliable response within individual participants across testing sessions, whilst line bisection showed very little reliability across session.

### 3.2 Hypothesis II: reliability across modality

**Fig. 7:**
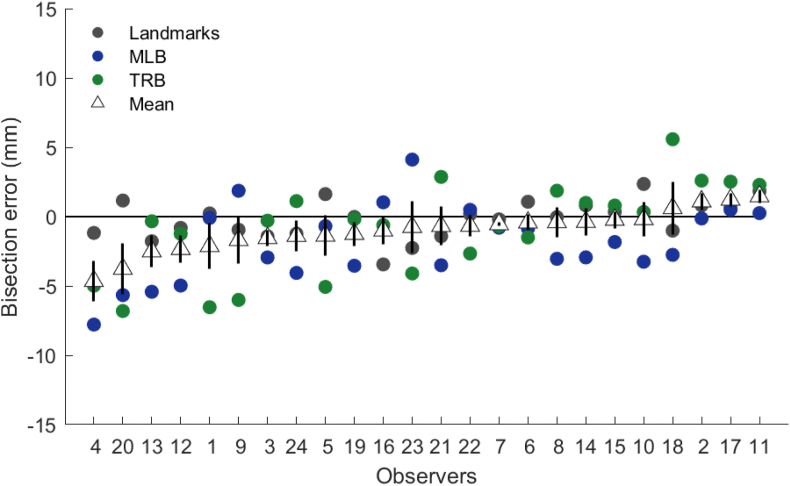
Individual bisection error for each task modality, collapsed across testing session. Data are ranked by bisection error from left (most negative) to right (most positive). Mean bisection error for each individual in each task represented by the white triangle. Error bars show standard error of the mean.

**Fig. 8:**
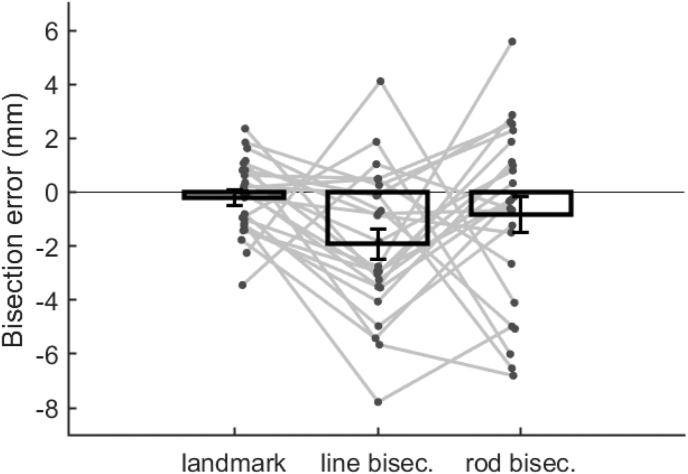
Summary of mean bisection error for each task (bars). Individual participant data for each task are displayed as grey dots. Error bars show standard error of the mean.

To address the hypothesis that error in bisection tasks is reliable within individuals across sensory modalities, bisection errors for each task were averaged across sessions and results were compared across task. Cronbach's α revealed no significant reliability across tasks (α = −0.12, *p* > .050), showing bisection error manifests differently across different bisection tasks within the same individual (Fig. 7). A left-ward bisection error was identified in each task, across all participants (Fig. 8), however the line bisection task produced the only significant left-ward error (t_23_ = −3.44, *p* = .002). No significant effect of pseudoneglect was found for either the landmark (t_23_ = −0.69, *p* > .050) or tactile rod bisection tasks (t_23_ = −1.24, *p* > .050). These results demonstrate clear differences in bisection error between tasks associated with different sensory modalities.

Pearson's r was also used to identify whether correlations in bisection error were present between two, out of the three tasks. We found no significant correlations between any of the tasks used. Results are presented in more detail in the Supplementary Materials.

### 3.3 Group differences between task and session

A repeated-measures ANOVA was conducted to identify any differences in bisection error between different testing sessions and tasks in the entire sample. The main effect of session was not significant (F_3,69_ = 2.62, *p* = .058, η^2^ = 0.10) and there were no significant main effects of task (F_3,69_ = 2.58, *p* = .087, η^2^ = 0.10), as well as no significant interaction effects. The results of the repeated measures ANOVA showed no significant differences in overall bisection error between the tasks and sessions.

## 4 Discussion

The overall aim of this study was to determine how reliable pseudoneglect is, within individuals, across sessions and modality. This was achieved by testing the reliability of response bias within the same individual to three different bisection tasks, line bisection, landmark and tactile rod bisection, across four separate testing sessions. We hypothesised that individual errors in bisection tasks would be reliable across 1) session and 2) task modality.

When data were averaged across both testing session and task, a significant effect of pseudoneglect was identified, supporting the majority of previous literature (Brooks et al., 2014; Friedrich et al., 2018; Jewell and McCourt, 2000). For each task and each testing session an average left-ward bisection error was also identified (Fig. 6, Fig. 8), however the only task to show significant effects of pseudoneglect was line-bisection, and session two was the only session to produce a significant mean effect of pseudoneglect. This does not concur with previous findings, as most studies identify significant left-ward biases for both landmark and tactile rod tasks (Benwell et al., 2014; Bowers and Heilman, 1980; Brooks et al., 2016; Jewell and McCourt, 2000; Learmonth et al., 2015; Milner et al., 1993). Tests of reliability within individuals suggests individuals may respond differently for each task. However, a repeated measures ANOVA showed no clear differences in bisection error observed across the group between task type or session.

### 4.1 Reliability across session

When we assessed reliability of response bias across four separate testing sessions, the landmark task showed strongest reliability in bisection error across each session, whilst moderate levels of reliability were identified for tactile rod bisection (Fig. 5). Bisection error for the line bisection task revealed little to no reliability across session within individuals. Highly reliable response bias across session in the landmark task supports previous findings, however, reliability for line bisection across four sessions is far lower than previously identified across two time-points (Learmonth et al., 2015; McCourt, 2001). One reason for this could be that the line bisection task has much higher individual variance than the landmark task, reducing the reliability of this measure. This is an important issue to consider when assessing for the presence of both pseudoneglect and its clinical counterpart, spatial neglect.

Line-bisection is often used to measure the severity of spatial neglect as this task is easy to administer at bedside. Line-bisection has been identified as possibly poor measure of spatial neglect before (Binder et al., 1992; Harvey et al., 1995; McIntosh et al., 2005; Sperber and Karnath, 2016). For example, neglect patients typically show a ‘reversal’ in their bisection error when asked to bisect particularly small (<2 cm) lines, and actually bisect these lines further to the *left*-hand side instead of the anticipated right side (Marshall and Halligan, 1989; Tegnér and Levander, 1991). The opposite pattern has since been identified in healthy individuals (Rueckert et al., 2002), emphasising how task-based factors can affect response to the line bisection task in the same individuals. The data from the present study shows that measurements from one session are inconsistent from measurements collected in a different session in the same neurotypical individual, as the observed Cronbach's α of −0.11 is a poor level of reliability to use for making inferences about an individual. The presence of pseudoneglect at the group level suggests that low reliability is due to the high within-participant variance observed. Future work on ways to reduce the within-participant variance could make an important contribution to the usefulness of the line bisection task. This result, along with previous findings, highlights the need for careful interpretation of bisection data in both healthy and clinical populations, and for any results to be accompanied by further neuropsychological testing.

Whilst the present study suggests poor test-retest reliability in the line-bisection task, it strongly supports the use of the landmark task to assess the presence of biases to spatial attention reliably across session. The Cronbach's α level of 0.80 is considered a good level of reliability to be able to use the results to make inferences about an individual. Despite the high response reliability, the landmark task produced the weakest mean group bisection error. This result is not consistent with the majority of previous literature that show a population effect of pseudoneglect in response to the landmark task (Benwell et al., 2013, 2014; Harvey et al., 1995; Heber et al., 2010; Learmonth et al., 2015). Unlike many studies, our work highlights the sometimes-large individual differences in bisection error in the landmark task, with some individuals showing a clear left-ward bias, whilst others were more biased towards the right (Fig. 5 top). This trend towards substantial individual differences in response bias has been identified previously within the literature in a number of studies (McCourt and Olafson, 1997; Slagter et al., 2010; Szczepanski and Kastner, 2013; Zago et al., 2017; McCourt, 2001) and could possibly explain why we see no effect of pseudoneglect here.

One example of such a study found bisection error to be significantly modulated by fronto-parietal activity in the contralateral hemisphere (Szczepanski and Kastner, 2013). Of the 7/12 participants showing a right-sided bias in the landmark task (opposite to pseudoneglect), 6 of them presented with significantly associated activity in their left hemisphere. This finding shows clear differences in how the brain responds to bisections tasks and highlights individual differences in the allocation of spatial attention (Reuter-Lorenz et al., 1990). Alongside this, the findings in the present study also suggest that there are individual differences in response across tasks, emphasising the need to assess individual differences in bisection error.

### 4.2 Reliability across modality

The present study used three different bisection tasks typically used to assess biases in spatial attention: the landmark (Harvey et al., 1995; Milner et al., 1993), line bisection (Axenfeld, 1915) and tactile rod bisection (Bowers and Heilman, 1980). Based on hypotheses on the origins of pseudoneglect (Heilman and Van Den Abell, 1979; Kinsbourne, 1970), we expected high reliability of response within individuals across modality. However, bisection errors within individuals showed no clear reliability across the three tasks (Fig. 7). This supports previous literature that also found no reliable effect of pseudoneglect across different visual pseudoneglect tasks (Learmonth et al., 2015), and different modalities (Brooks et al., 2016; Luh, 1995). This suggests that supra-modal attentional mechanisms may not be responsible for creating the observed biases in pseudoneglect.

These findings have important implications, as two of the most common ways to assess the presence of pseudoneglect is through either the line-bisection or the landmark task and the presentation and results of these two tasks are often used interchangeably. This flexible use of different bisection tasks relies on the idea that pseudoneglect presents itself similarly within individuals across these three tasks. However the work in the present study supports previous findings in both pseudoneglect (Brooks et al., 2016; Learmonth et al., 2015; Luh, 1995; Nicholls et al., 1999) and spatial neglect (Monika Harvey et al., 2002). Harvey et al. (2002) found evidence of both perceptual and visuomotor biases in spatial neglect, yet upon further examination, the magnitude of perceptual and visuomotor bias were remarkably different. These results highlight an important distinction between tasks that assess the presence of spatial biases: different bisection tasks can produce different response biases in the same individual. Better distinctions need to be made between tasks used to assess pseudoneglect and spatial neglect, with careful consideration given to how using a particular bisection task might affect biases in a particular sample. As each task used in the present study taps into slightly different modalities, it is possible that different sensory modalities produce differing response biases within individuals.

This finding is somewhat supported by neuroimaging studies. A range of functional MRI studies have shown how different regions of the brain respond to different attention-based tasks (Binder et al., 1992; Molenberghs et al., 2012; Revill et al., 2011; Zago et al., 2017); however functional differences between different bisection tasks has rarely been investigated. One of the few studies that has done this found an overlapping network of regions in the parietal lobe associated with both the line bisection and landmark task (Cicek et al., 2009). However, they also found regions that were independently associated with each task, highlighting functional differences between the two tasks. The lack of reliability found between bisection tasks in the present study fits with this result and lends support to the idea that biases in spatial attention in these tasks are driven by independent neural networks. An interesting follow up from this work would be to use neuroimaging to determine the distinct processing regions for the tactile-rod bisection task, and whether there are any regions of overlap between landmark and line-bisection tasks for this third, lesser known bisection task.

### 4.3 Conclusions

The essence of this work was to further our understanding of the implicit spatial biases that are present among the general population and found evidence towards individual variation in pseudoneglect. Individual differences in bisection error has been identified previously (Nicholls et al., 1999; Zago et al., 2017), however the proportion of individuals who show pseudoneglect in the general population is yet to be studied. A promising follow-up from this study would be to investigate the spread of individual differences in response bias within the general population, to gain insight into the expected distribution of bias as well as the overall prevalence of pseudoneglect. Further study of neurotypical individuals that fall at the extreme ends of the distribution could provide insight into the cognitive and neurological underpinnings of what drives biases of spatial attention. Moreover, understanding individual differences in spatial attention and the differences between tasks might inform our knowledge of the heterogeneities behind the presentation of neglect symptoms (Vaessen et al., 2016).

Whilst the results of this study reveal insights into overall reliability of pseudoneglect, we are unable to offer a causal explanation for this bias. As biases within individuals are not the same for each modality, it is likely that the source of bias is different too. At first glance, this does not support attention hypotheses of pseudoneglect such as right-hemisphere dominance (Heilman and Van Den Abell, 1979) and interhemispheric competition (Kinsbourne, 1970), as these attempt to explain the presence of pseudoneglect across sessions and tasks. Poor intra-task validity identified in this study leads to important questions about the nature and cause of pseudoneglect, and possibly spatial neglect. It is possible that a one-fits-all account of pseudoneglect might be over-simplifying our understanding of the mechanisms behind the orienting of spatial attention across modalities. Future work directly investigating these individual and task differences in both pseudoneglect and spatial neglect, as well as patterns of modality-specific response will help to better our understanding of the source of implicit biases to spatial attention.

## CRediT authorship contribution statement

**A.G. Mitchell:** Conceptualization, Investigation, Methodology, Software, Resources, Validation, Formal analysis, Writing - original draft, Writing - review & editing, Visualization. **J.M. Harris:** Conceptualization, Methodology, Validation, Resources, Writing - review & editing, Supervision, Funding acquisition. **S.E. Benstock:** Methodology, Investigation, Formal analysis. **J.M. Ales:** Conceptualization, Methodology, Software, Resources, Formal analysis, Writing - review & editing, Supervision, Funding acquisition.

## Declarations of competing interest

The authors declare that they have no known competing financial interests or personal relationships that could have appeared to influence the work reported in this paper.
