# Supplementary material for: The reliability of pseudoneglect is task dependent
Source: Neuropsychologia. 2020 Nov;148:107618. doi: 10.1016/j.neuropsychologia.2020.107618 (PMC7718110; doi:10.1016/j.neuropsychologia.2020.107618)
Supplement: Multimedia component 1 [file mmc1.docx]

**The reliability of pseudoneglect is task dependent – Supplementary Materials**

*Mitchell, A. G^1,2^, Harris, J. M^2^, Benstock, S^2^, Ales, J. M^2^*

**Exploratory analyses**

1. **Reliability analysis for left- and right-hand responses**

As we used both left- and right-hand responses for both manual line bisection and tactile rod bisection tasks, it is possible that reliability for these tasks was compromised by the differences in response that typically occur between right and left hands (Bradshaw et al., 1986; Brodie & Pettigrew, 1996). To address this possibility, we analysed bisection error for these two tasks for the left- and right-hand responses separately. As in previous analyses, Cronbach’s alpha was used to assess reliability across session and modality (task). The results are reported below.

- 1. *Reliability across session*

For the manual line bisection task, mean bisection error for left-hand responses was -2.05mm (SD = 2.62mm) and -1.79mm (SD = 3.09mm) for right-hand responses. Cronbach’s alpha showed poor reliability in bisection error across all four testing sessions for both left-hand (α = 0.19, *p* = .249) and right-hand responses (α = .05, *p* = .411). Both left- and right-hand responses revealed left-ward shifts in the perceived midpoint but splitting results by hand did not increase the reliability of this task across session.

For the tactile rod bisection task, mean bisection error for left-hand responses was 2.03mm (SD = 3.88mm) and -3.69mm (SD = 5.22mm) for right-hand responses. Both left-hand (α = 0.61, *p* = .001) and right-hand (α = 0.61, *p* = .002) conditions showed moderate, but significant reliability in bisection error across time. However, reliability for either hand was not higher than overall reliability observed for both hands (α = 0.63, *p* < .001), as reported in the manuscript.

*1.2 Reliability across modality*

A moderate negative Cronbach’s alpha revealed opposite responses across each task for the left-hand (α = -0.53, *p* = .001) whilst reliability for right-hand bisection error was poor (α = 0.02, *p* = .462). Reliability across task modality does not increase if responses are split across left and right hands.

*1.3 Reliability across hand*

To see whether hand used significantly affected reliability of response we used Cronbach’s alpha to compare bisection error across both hands in manual line bisection and tactile rod bisection.

For manual line bisection reliability in bisection error across left and right hands was high (α = .90, *p* < .001), however this was not the case for tactile rod bisection, where reliability across hand was weak (α = .05, *p* = .452). Splitting bisection error into right- and left-hand responses did not improve reliability across tasks.

1. **Effect of colour reversal on perceived midpoint in landmark task**

We also investigated the possible effect of colour reversal, indicating the ‘midpoint’ on lines in the landmark task (Figure S1), on perceived midpoint of the line. It is possible that the contrast change between white and black portions of the lines shifted perception of midpoint further to the left or right, reducing likelihood that pseudoneglect is detected. As both line configurations were presented an equal number of times in each session, any possibility of this illusion effecting reliability of response across sessions is highly unlikely, therefore this analysis focuses on differences in average response across participants between the two line configuations.


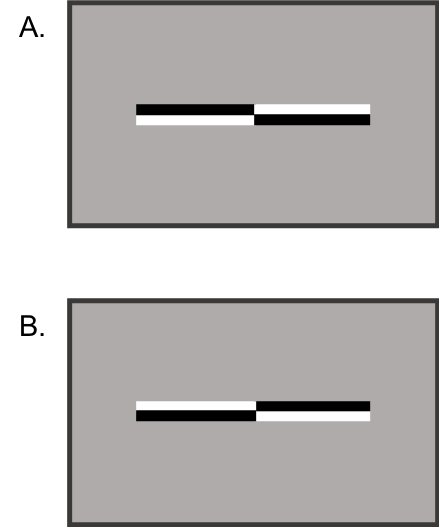


Figure 1: Possible white-black configurations of lines in the landmark task (A) configuration 1 = black top-left and (B) configuration 2 = white top-left.

Psychometric functions were fitted to right-side longer responses for each line configuration: (1) top left black (Figure S1A) and (2) top left white (Figure S1B)*.* Next, we extracted the mean point of subjective equality (PSE, the point at which the line was perceived to be bisected at the midpoint, full details described in methods of main paper, *Section 3.4.1ii)* and used a t-test to compare bisection error for each line configuration;

A repeated-measures t-test revealed no difference (t_24_ = 0.47, *p* = .639) in the PSE, therefore bisection error, between line configuration (1) - top left black (mean = 0.12mm, SD = 1.72mm) and line configuration (2) – top left white (mean = -0.03mm, SD = 2.02).

1. **Reliability across modality – Pearson’s correlations**

In the main paper, Cronbach’s alpha found weak reliability in bisection error across three tasks; landmark, line bisection and tactile rod bisection. As Cronbach’s alpha is an aggregate measure of all three tasks it is possible that correlation between a subset of the tasks went undetected. In addition, as Cronbach’s alpha is designed to treat measurements on the same scale it specifically does control for cross-measure variance. In order to address these two issues, and to prise apart the relationship between the three tasks, we used Pearson’s r to assess correlation in bisection error between; landmark vs. line bisection, landmark vs. tactile rod bisection and line bisection vs. tactile rod bisection.

We found that bisection error was not correlated between landmark and line-bisection (r = -0.02, *p* = .911), landmark and tactile rod bisection (r = 0.00, *p* = .996) or manual line bisection and tactile rod bisection (r = -0.07, *p* = .732). These results confirm the findings presented in the main paper, that there is no within individual reliability in different bisection tasks that assess pseudoneglect.
